# A surface electromyography and inertial measurement unit dataset for the Italian Sign Language alphabet

**DOI:** 10.1016/j.dib.2020.106455

**Published:** 2020-10-22

**Authors:** Iacopo Pacifici, Paolo Sernani, Nicola Falcionelli, Selene Tomassini, Aldo Franco Dragoni

**Affiliations:** Dipartimento di Ingegneria dell'Informazione, Università Politecnica delle Marche, Via Brecce Bianche 12, 60131 Ancona, Italy

**Keywords:** Italian Sign Language, Electromyography, EMG, Inertial Measurement Unit, IMU, Gesture Recognition, Myo Armband

## Abstract

Surface Electromyography (EMG) and Inertial Measurement Unit (IMU) sensors are gaining the attention of the research community as data sources for automatic sign language recognition. In this regard, we provide a dataset of EMG and IMU data collected using the Myo Gesture Control Armband, during the execution of the 26 gestures of the Italian Sign Language alphabet. For each gesture, 30 data acquisitions were executed, composing a total of 780 samples included in the dataset. The gestures were performed by the same subject (male, 24 years old) in lab settings. EMG and IMU data were collected in a 2 seconds time window, at a sampling frequency of 200 Hz.

## Specifications Table

**Table:** 

Subject	Signal processing
Specific subject area	Gesture Recognition, Touchless Interaction
Type of data	Json Text Files (.json)
How data were acquired	The dataset was collected using the raw data from the Myo Gesture Control Armband Technical specifications: https://web.archive.org/web/20200528111822/https://support.getmyo.com/hc/en-us/articles/202648103-Myo-Gesture-Control-Armband-tech-specs
Data format	Raw
Parameters for data collection	The dataset includes surface electromyography (EMG) signals from 8 sensors and Inertial Measurement Unit (IMU) data, i.e. orientation, acceleration, gyroscope, for the gestures representing the 26 letters of the Italian Sign Language alphabet. Each gesture sample was recorded in a two seconds time window, using a sampling frequency of 200 Hz. All the gestures were performed by the same subject (male, 24 years old) in lab settings.
Description of data collection	For each gesture representing a letter, 30 acquisitions were performed, composing a total of 780 acquisitions. The Myo Armband was placed on the right arm, always in the same position. The dataset is therefore organized into 26 directories, one for each letter of the alphabet. Each directory includes 30 json files, one for each acquisition. Each json file includes: - 400 8-dimensional vectors, i.e. the series of values of the 8 EMG sensors; - 400 3-dimensional acceleration vectors - 400 4-dimensional orientation vectors, i.e. the quaternion of the orientation - 400 3-dimensional gyroscope vectors
Data source location	Dipartimento di Ingegneria dell'Informazione, Università Politecnica delle Marche, Ancona, Italy.
Data accessibility	Public repository: Mendeley Data Repository name: An EMG and IMU Dataset for the Italian Sign Language Alphabet Data Identification Number: 10.17632/dzgczt98cz.1 Direct URL to data: http://dx.doi.org/10.17632/dzgczt98cz.1

## Value of the Data

• As surface EMG and IMU sensors have been proven useful for sign language recognition, the dataset is an open and easy to download resource to develop and test related algorithms. Even if the dataset includes gestures from the Italian Sign Language, algorithms results can be generalized to other sign languages.
• Researchers involved in gesture recognition and automatic recognition of sign language can use the data to benchmark their algorithms. In the long term, end-users who need to interpret sign language gestures can benefit from applications built and tested on the data (e.g. applications for real-time translation).
• The dataset can be used to train and test deep learning models for automatic gesture recognition or, in general, gesture recognition algorithms. Moreover, data augmentation techniques can be developed and/or tested on the provided data.

## 1 Data Description

**Fig. 1:**
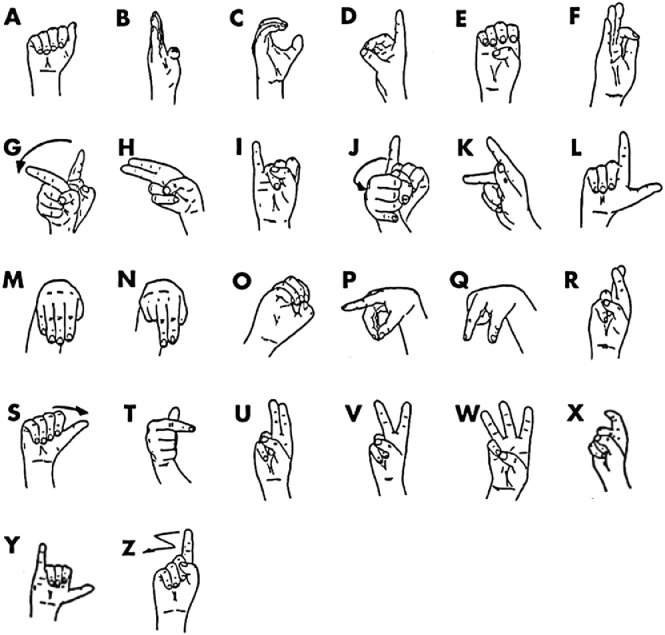
The gestures of the Italian Sign Language alphabet.

Gesture recognition has a plethora of application domains, including, for example, human-robot collaboration [1,2], rehabilitation [3] and touchless interaction with smart objects [4]. In recent years, gesture recognition based on surface Electromyography (EMG) and Inertial Measurement Units (IMU) has gained attention for the automatic detection of sign languages gestures [5,6,7]. To this end, we present a dataset of EMG and IMU data of the gestures of the Italian Sign Language alphabet (Fig. 1). The gestures were collected using the Myo Gesture Control Armband^1^.

The dataset contains 780 gesture acquisitions (30 for each letter of the alphabet) and is organized as follows:

• Data is split into 26 directories, one for each letter of the alphabet. Therefore, a directory includes all the 30 gesture acquisitions of a single letter, identified by the name of the directory.
• Each directory includes 30 json file, one for each acquisition of the gesture representing a letter.
• Each json file is named using a Global Unique Identifier (GUID). It includes the data from the 8 EMG sensors and the IMU of the Myo Armband, collected during a single acquisition of the gesture in a 2 seconds time window, at a sampling frequency of 200 Hz.

**Fig. 2:**
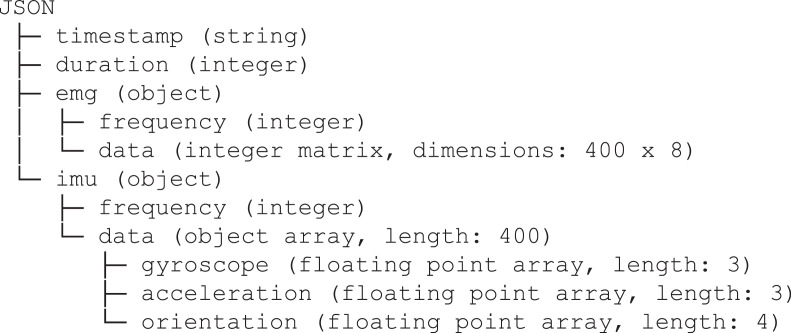
The structure of each json file containing the data collected in a single gesture acquisition.

Fig. 2 shows the structure of a single json file. Each json file contains a json object with the following fields:

• timestamp, a string representing the date and time of the gesture acquisition. For example, the string “09/07/20/10:03:19” suggests that the gesture and its acquisition were performed the 9^th^ of July 2020, at 10:03:19 a.m.;
• duration, an integer describing how long the data acquisition of the gesture was, in milliseconds. The value is 2000 in all the json files, since the dedicated time window was 2 seconds for each acquisition;
• emg, an object representing the EMG data of the gesture. It has two fields
  ○ frequency, i.e. the sampling frequency (in Hz) of the values from the EMG sensors. This value is 200 in all the json files;
  ○ data, a 400 × 8 integer matrix. Each row is then an 8-dimensional array including the values from the 8 EMG sensors of the Myo Armband. Therefore, data is the time series of the values from the EMG sensors during the acquisition of the gesture;
• imu, an object representing the IMU data of the gesture acquisition. It has two fields
  ○ frequency, i.e. the sampling frequency (in Hz) of the values from the IMU. This value is 200 in all the json files;
  ○ data, a 400 elements length object array. Each object has three fields, namely gyroscope (an array composed by 3 floating point values), acceleration (an array composed by 3 floating point values), and rotation (an array composed by 4 floating point values).

## 2 Experimental Design, Materials and Methods

Each gesture acquisition was performed by the same person (male, 24 years old) wearing the Myo Armband on his right arm, always in the same position. Each gesture acquisition was done for 2 seconds, sampling both EMG and IMU data at 200 Hz. The values from the 8 EMG sensors and the IMU are the raw data provided by the Myo Armband SDK. Thus, a total of 780 gestures were collected and included in the dataset (30 for each letter of the alphabet).

**Fig. 3:**
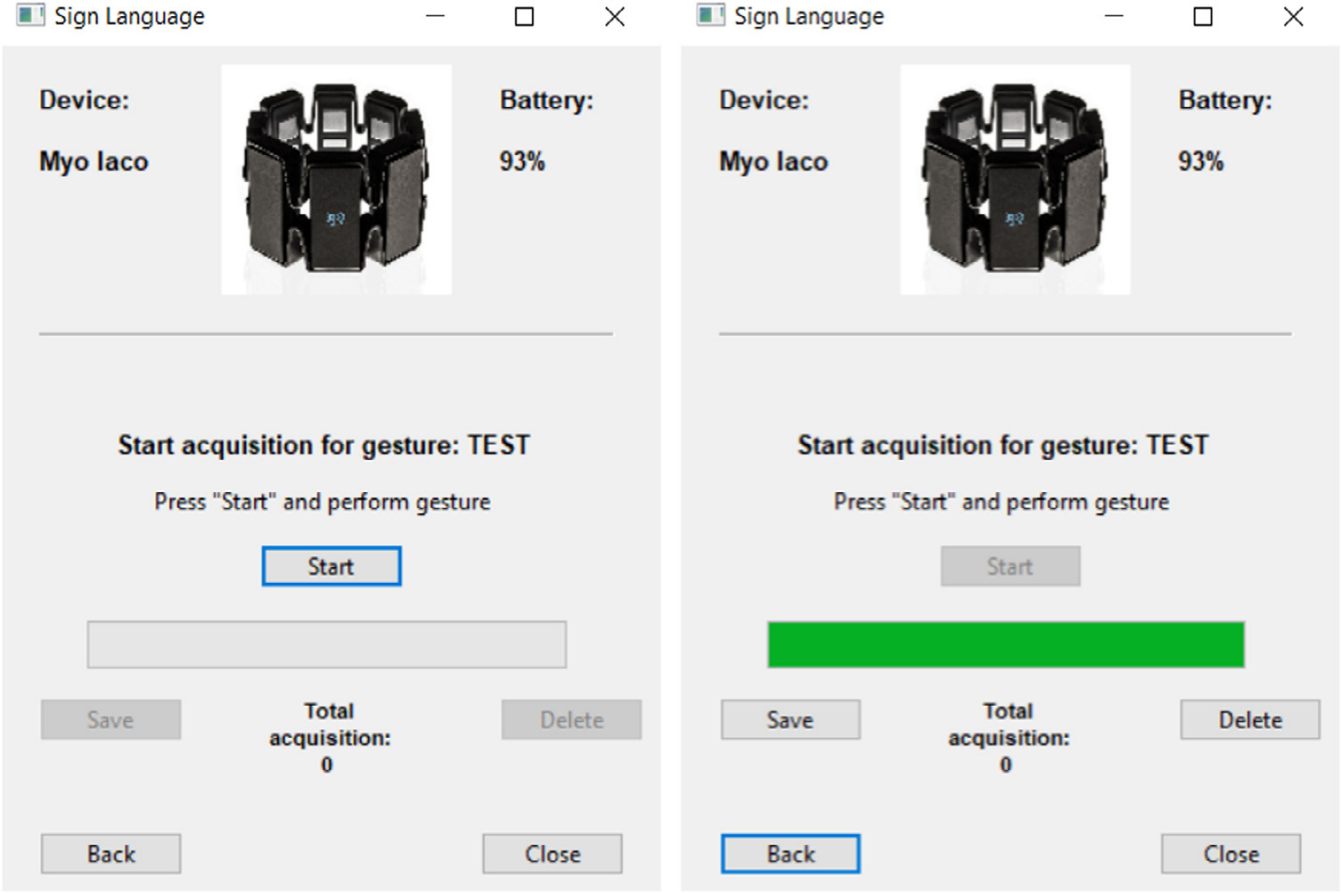
The app developed to collect gesture data, before (left) and after (right) the gesture acquisition. The source files are available in the data repository.

Each gesture was self-collected by the same subject executing it, using the dedicated app that we developed on purpose^2^. As showed in Fig. 3, the subject is supposed to press “start” when ready to execute the gesture. The acquisition duration is highlighted by a progress bar.

The data included in the dataset can be augmented with various techniques, including time-warping, scaling, jittering [8]. Among those, the rotation of sensors seems the most promising in terms of accuracy when applied [9]. Intuitively, it simulates gestures performed wearing the sensors (i.e. the Myo Armband) at different rotations.

## Ethics Statement

The authors received informed consent from the participant who recorded the gestures in the dataset. The data uploaded in the public repository does not identify the participant, as it includes only values from Electromyography and IMU sensors without any personal data.

## Declaration of Competing Interest

The authors declare that they have no known competing financial interests or personal relationships which have, or could be perceived to have, influenced the work reported in this article.

## Footnotes

1 https://web.archive.org/web/20200528111822/https://support.getmyo.com/hc/en-us/articles/202648103-Myo-Gesture-Control-Armband-tech-specs

2 The source code is available on GitHub at https://github.com/airtlab/An-EMG-and-IMU-Dataset-for-the-Italian-Sign-Language-Alphabet
